# Beyond Conventional Coronary Aspiration: Advances in Thrombus Management

**DOI:** 10.1016/j.jaccas.2026.108977

**Published:** 2026-07-15

**Authors:** Rustem Dautov, Soleiman Aria

**Affiliations:** a Heart and Lung Institute, The Prince Charles Hospital, Brisbane, Queensland, Australia; b School of Medicine, University of Queensland, Brisbane, Queensland, Australia

**Keywords:** myocardial infarction, percutaneous coronary intervention, thrombus aspiration

**Figure:**
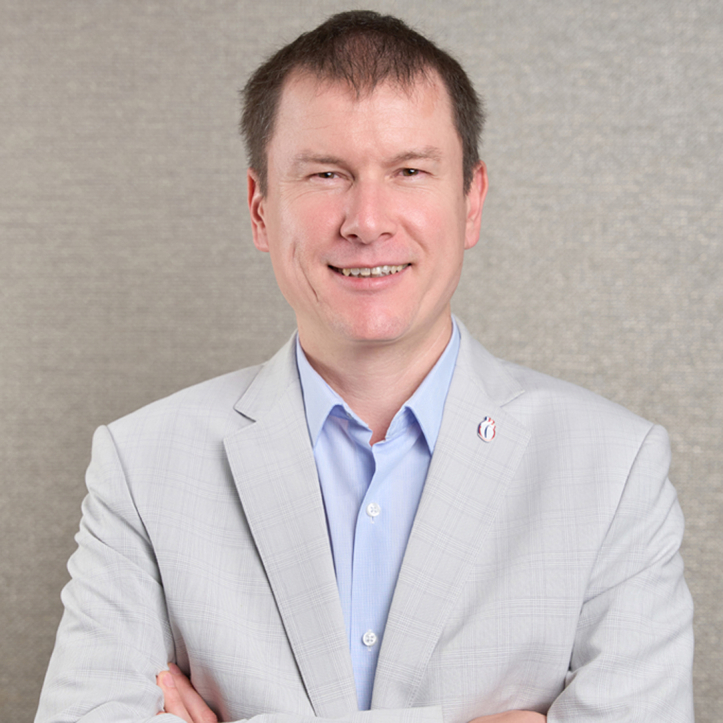

Primary percutaneous coronary intervention (PCI) is the gold standard reperfusion strategy for ST-segment elevation myocardial infarction (STEMI). However, a massive intracoronary thrombus remains a major challenge in interventional cardiology. Large thrombus burden is associated with distal embolization, no-reflow phenomenon, impaired myocardial perfusion, stent thrombosis, larger infarct size, and increased mortality.^1^^,^^2^ Despite advances in pharmacotherapy and device technology, optimal management remains controversial. However, evolving aspiration technologies have reignited interest in selective thrombus extraction in carefully chosen patients.

Manual aspiration thrombectomy gained widespread adoption after early studies such as TAPAS suggested a survival benefit.^3^ However, larger randomized trials including TASTE and TOTAL failed to demonstrate a mortality benefit with routine thrombectomy and raised concerns regarding increased stroke risk.^4^^,^^5^ Consequently, current European Society of Cardiology and American College of Cardiology guidelines no longer recommend routine thrombus aspiration during primary PCI, although selective or bailout use may still be considered in cases of large residual thrombus burden or failed reperfusion.^1^^,^^2^ A question remains as to why thrombectomy fails to improve clinical outcomes. It has been proposed, based on intracoronary optical coherence tomography findings from a TOTAL trial substudy,^6^ that thrombectomy does not significantly reduce culprit-lesion thrombus burden compared with PCI alone.^7^

It is important to note that the earlier trials largely evaluated older-generation manual aspiration catheters and did not fully encompass newer aspiration systems currently available. Recent years have seen the emergence of more advanced aspiration technology designed to improve extraction efficiency while minimizing distal thrombus embolization. These technologies have evolved along 2 parallel pathways. The first involves refinement of conventional coronary aspiration catheters with the newer devices incorporating larger extraction lumens and improved deliverability within tortuous coronary anatomy. The second pathway has emerged through adaptation of neurointerventional aspiration technology originally developed for acute ischemic stroke thrombectomy. Examples of both aspiration catheter categories, also detailed below, are provided in Table 1.

**Table 1:** Technical Specifications of Currently Available Coronary Aspiration Catheters

No	Aspiration catheter	Diagram	Product Details
1	Medtronic Export Adv www.medtronic.com		Manual syringe Preloaded stylet enhances shaft stiffness, improving trackability and pushability Extraction lumen size of 0.044 inches proximally and 0.043 inches distal Compatible with a 6-F guide catheter
2	Thrombuster III SL www.kaneka-med.jp		Manual syringe Enhanced antikinking property by a braid structure Extraction lumen size of 0.039 inches or 0.049 inches Compatible with 6-F or 7-F guide catheter
3	ReBirth Pro 2 www.nipro.co.jp		Manual aspiration Has extended 22-cm guidewire lumen that improves guidewire tracking and deliverability Extraction lumen size of 0.044 inches or 0.049 inches Compatible with a 6-F or 7-F guide catheter
4	Penumbra Indigo CAT RX www.penumbrainc.com		Penumbra continuous aspiration pump Enhanced deliverability with a proximal laser-cut hypotube Extraction lumen size of 0.044 inches Compatible with a 6-F guide catheter
5	Sofia Flow Plus www.terumoneuro.com (Off-label coronary use)		Compatible with manual and pump aspiration Hybrid coil and braid reinforcement offers enhanced kink resistance and maintains aspiration lumen Extraction lumen size of 0.070 inches Compatible with a 6-F guide catheter
6	Lawnest Wu et al 2026^8^		Self-expanding, stent-like, retrieval system Used stand-alone or in combination with aspiration catheters (eg, ReBirth as in this example) Compatible with 6-F guide catheters

The Export (Medtronic) catheters have historically been among the most widely used thrombectomy devices in STEMI intervention, including in the landmark TAPAS, TASTE, and TOTAL trials. The Thrombuster (Kaneka) aspiration catheters also remain widely used due to their favorable deliverability profile. It is more commonly used in Japan and the Asian-Pacific region. More recently, the ReBirth (Nipro) catheter has emerged as another aspiration platform designed to enhance thrombus extraction by being more hydrophilic than the former 2 catheters. Although these systems are straightforward and rapidly deployable, their efficacy may be limited in the presence of organized and exceptionally large thrombus burden.^9^

Recently, more advanced continuous vacuum aspiration systems originally developed for neurointervention have been adapted for coronary thrombectomy. The Penumbra's Indigo CAT RX aspiration system (Penumbra Inc) comprises the CAT RX aspiration catheter and a Penumbra aspiration pump, which provides a continuous vacuum aspiration. Although it possesses a comparable aspiration lumen, it is potentially a superior thrombus removal device to syringe-based manual aspiration systems. Early observational data have demonstrated promising procedural success in patients with massive coronary thrombus.^10^^,^^11^

The Sofia catheter (Microvention), originally designed for neurointervention, has been innovatively used in the management of complex coronary thrombus, albeit off-label. Its highly trackable distal segment facilitates delivery through tortuous coronary anatomy, while its large 0.070-inch extraction lumen exceeds that of other currently available aspiration catheters, making it an attractive bailout option when other aspiration systems fail. Case reports have described successful utilization of the Sofia aspiration catheter where conventional coronary thrombectomy devices failed to restore adequate coronary flow.^12^^,^^13^

In this issue of *JACC: Case Reports*, Wu et al^8^ report the use of a novel thrombus retrieval device, The Lawnest (APT Medical).^8^ First introduced by Yang et al^14^ in 2025, the device represents a coronary-specific bailout strategy for refractory thrombus when conventional aspiration thrombectomy fails. Unlike standard aspiration systems, the Lawnest functions as a stent-like retrieval device and can be used with 6-F aspiration catheters. Constructed from nitinol, the device is self-expanding, eliminating the need for balloon deployment and allowing atraumatic adaptation to the coronary vessel while minimizing thrombus disruption during the retrieval. A conceptually similar neurointerventional stent retriever approach was first described in the coronary circulation in 2017 using the Solitaire device (Medtronic) for left main coronary artery thrombectomy.^15^ Wu et al^8^ integrated the Lawnest device with the ReBirth aspiration catheter through a hemostasis Y-valve, allowing continuous negative pressure during thrombus retrieval. This hybrid system enabled simultaneous en-masse mechanical thrombus capture and continuous aspiration, potentially reducing the risk of distal embolization. Similarly, a paper by Chong et al^16^ describes successful management of a massive intracoronary thrombus using another hybrid thrombectomy system combining the EnVast (Vesalio) stent retriever in combination with the Penumbra's CAT RX aspiration system.

Mechanical thrombectomy should be combined with aggressive pharmacological therapy. Unfractionated heparin remains fundamental during primary PCI by reducing thrombin-medicated clot propagation and catheter thrombosis. Glycoprotein IIb/IIIa inhibitors, including abciximab, tirofiban, and eptifibatide, continue to have a role in selected high thrombus burden STEMI cases by inhibiting platelet aggregation and can be administered intravenously and intracoronary. Dual antiplatelet therapy with aspirin and a potent P2Y12 inhibitor such as ticagrelor, prasugrel, or cangrelor remains essential. In extreme thrombotic burden, prolonged intravenous antithrombotic therapy and deferred stenting may reduce no-flow and acute stent thrombosis.^17^

Ultimately, management of coronary thrombus in STEMI requires a nuanced and individualized strategy integrating pharmacological therapy, mechanical thrombectomy, and intravascular imaging where appropriate. Although routine aspiration thrombectomy has fallen out of favor, innovation continues to reshape therapeutic options in one of interventional cardiology's most challenging scenarios. Contemporary aspiration platforms provide larger extraction lumens, continuous suction systems, en-masse mechanical capture, and improved catheter deliverability compared with devices used in earlier routine thrombectomy trials. As a result, selective aspiration may re-emerge as a targeted rescue strategy in patients with massive thrombus burden. Importantly, evidence derived from older devices cannot necessarily be extrapolated to newer technologies. Future evidence should therefore evolve sequentially through case reports, case series, observational studies, randomized control trials, and meta-analyses as the quest to improve interventional cardiology continues.

## Funding Support and Author Disclosures

The authors have reported that they have no relationships relevant to the contents of this paper to disclose.
